# Expectations of employers in the United States for entry-level public health job skills with a bachelor’s degree: an analysis of the positions advertised in an online job portal

**DOI:** 10.3389/fpubh.2023.1218509

**Published:** 2023-10-05

**Authors:** Satish K. Kedia, Coree Entwistle, Guijin Lee, Laura Magaña, Emily M. Burke, Ashish Joshi

**Affiliations:** ^1^School of Public Health, University of Memphis, Memphis, TN, United States; ^2^Association of Schools and Programs of Public Health (ASPPH), Washington, DC, United States

**Keywords:** public health, education, employment, job skills, entry-level, bachelor’s degree

## Abstract

**Objectives:**

To analyze the current public health labor market for bachelor’s-level graduates.

**Methods:**

Public health-related job postings (*N* = 365) from across the United States were collected from an online job portal, Indeed.com, from November 7–14, 2022. Job titles, organization type, degree requirements, work experience, job location, and preferred skills for bachelor’s-level public health positions were analyzed.

**Results:**

Thirty-one job titles were identified. Approximately one-third (32.33%) of postings were from the Northeastern US; 23.56% were from the Southeastern region. Thirty-five job skill categories were identified. Most jobs (92.33%) required oral and written communication skills, and 85.21% specified educational skills for public health promotion. Cultural competency, project management, and case management abilities were also highly sought.

**Conclusion:**

This study revealed the needs of the public health workforce and bolstered the case that public health degree-seekers should be equipped with a set of strategic skills applicable to a range of multisectoral and multidisciplinary public health jobs.

**Policy implications:**

Given the rapid changes in the field of public health, ongoing analysis of the labor market benefits educators, employers, and policymakers alike.

## 1. Introduction

Even before the COVID-19 pandemic, demands for public health interventions were on the rise (1). The aging of the baby boomer generation (2), the looming threats of climate change (3), and the COVID-19 pandemic-related need for immunizations, testing, and reliable public health information have accentuated the demand for a well-trained public health workforce (4–6). Moreover, the pandemic led to elevated levels of professional burnout in many health- and public health-related jobs (7). Given the evolving conditions of community health, it behooves public health educators to consider workforce demands and synchronize public health higher education curricula with current and projected employer and community needs.

The World Health Organization (WHO) predicts a global healthcare worker shortage in the coming decade (8). Similarly, the U.S. Bureau of Labor Statistics projects that the nation’s healthcare and social assistance sectors will add approximately 2.6 million new jobs between 2021 and 2031, a rate of increase that exceeds any other workforce sector (2). Enrollments in public health degree programs at the undergraduate level are also currently on the rise. Over 36% of conferred public health degrees were undergraduate, and 57% were master’s degrees in the 2019–2020 academic year (9). Plepys et al.’s (10) study of over 53,000 graduates of Council on Education of Public Health (CEPH) accredited schools and programs showed that 73% of all graduates were employed, 15% had gone for further education, and 5% were engaged in internships within a year of graduation. However, while a higher number of individuals are being trained in public health, the question remains as to whether their training is adequate for current and future workforce demands.

Scholars have observed the shifting trends in public health and strive to identify the skills necessary for the public health workforce to meet the changing needs of our time (11–15). A report from the National Consortium for Public Health Workforce Development (13) compiled a list of specialized and strategic skills needed by public health workers. The six specialized skills were: communicable disease control, chronic disease and injury prevention, environmental public health, epidemiology, specific population focus (i.e., maternal, child, and family health, or LGBTQ populations), and health education. The report also identified eight strategic skills that are necessary regardless of any specialized focus: systems thinking, change management, persuasive communication, data analytics, problem solving, diversity and inclusion, resource management, and policy engagement (13). Most studies, as well as the Consortium report, acknowledge that training and education for public health have focused largely on specialized skills, leaving many workers with gaps in strategic skills (11, 13, 16).

An examination of deficits in the current public health workforce indicates that many public health workers who are not trained in public health informatics (PHI) feel underprepared to work with health informatics data (17). Similarly, McCullough’s (18) analysis of the 2017 Public Health Workforce Interest and Needs Survey (PH WINS) data showed skill gaps in financial proficiency among non-supervisory employees, especially in county-level health departments. Finally, health equity and social determinants of health (SDH) are being recognized locally and globally as critical components of effective public health services (19, 20). However, the focus on health equity and knowledge of SDHs, including health informatics, framed in terms of health equity, are not being translated into actionable efforts (21, 22).

The relationship between public health workforce skill demands and educational needs has been the focus of several surveys and studies, many of which are derived from either the 2017 or 2021 PH WINS data collected from government public health employees (23–25). Taylor and Yeager’s (16) study reported that public health workers without formal public health education experienced more core competency gaps than their co-workers with public health degrees. In addition, Treviño-Reyna et al. (6), who gathered European public health graduate and employee data, found that even highly educated and motivated public health workers faced poor working conditions and unacceptably low compensation, which created instability in appropriate crisis response, such as experienced during the COVID-19 pandemic. Krasna et al.’s (8) analysis focused specifically on workforce demand for public health graduates at the MPH level and determined that labor market demand outside the public health field may be compounding the shortage of public health workers. Other published studies had a sub-disciplinary focus, such as McLane et al.’s (12) or Joshi et al.’s (26) examination of health informatics employment, which identified the growing need for informatics training in the public health workplace. Our study has uniquely focused on entry-level job postings across public and private workforce sectors in the US, requiring a bachelor’s degree. This focus will allow educators and policymakers to make informed choices about the undergraduate curricula needed to meet the skill demands of the public health workforce.

## 2. Methods

**Figure 1:**
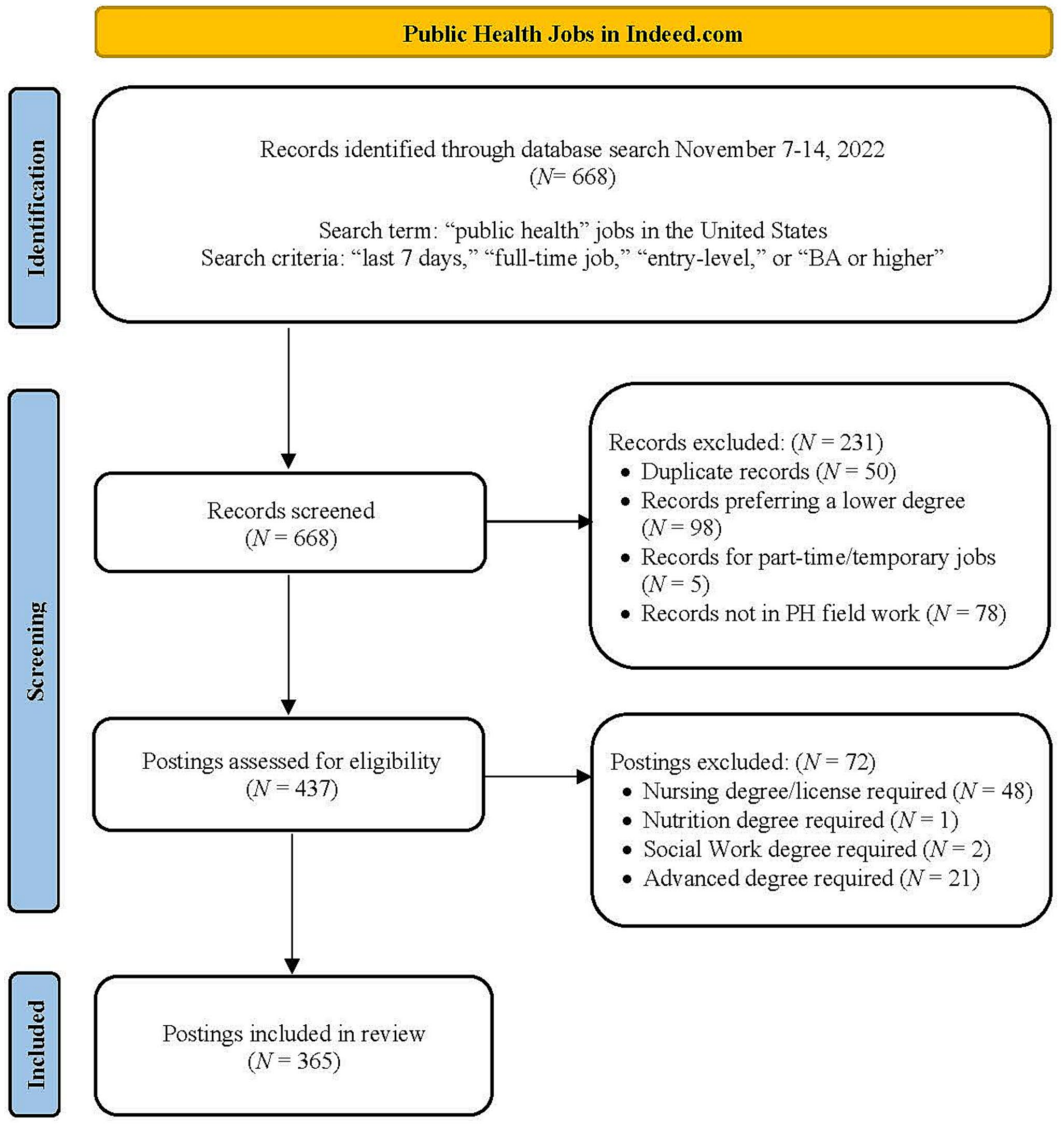
Search flow chart.

For our current study, data on all public health jobs advertised on Indeed.com were collected from November 7–14, 2022. Indeed.com is a common job portal in the U.S. and was chosen because it is the most trafficked online job portal in the country. Indeed.com aggregates millions of job postings across company career sites, job boards, staffing firms, and other online sources (26) and provides options for job posting search criteria. We restricted our search terms to “public health,” “last 7 days,” “full-time,” “entry-level,” and/or “bachelor’s degree” for better selection. The search was limited to jobs in the United States. The postings were reviewed by the first three co-authors, screening independently using the defined search term and criteria. The initial search for jobs in public health between November 7–14, 2022, returned 668 matches. After removing duplicates not meeting the criteria (i.e., lower than bachelor’s degree preferred, part-time jobs), and not public health-related positions (e.g., a driver in a community health center, a housekeeper in a health facility), 437 job postings remained. Excluding postings that required other degrees or licenses (e.g., nursing, nutrition, social work, etc.), the final selection resulted in 365 job postings (Figure 1).

The job postings that met the consensus of reviewers were extracted into an Excel spreadsheet. After finalizing the list, the first author double-checked all postings based on the search term, search criteria, and consensus rules. The data extraction included the following:

- Job title: Job titles from the postings were recorded and re-coded into similar job categories. For example, COVID-19 community health equity navigator, community health outreach, harm reduction outreach specialist, or youth outreach coordinator were categorized into the “Community Health Outreach Specialist.”
- Organization type: The types of organizations making the job postings were recorded and coded into 12 categories: (1) government, (2) non-profit organizations (NPO), (3) educational institution, (4) hospital, (5) healthcare provider, (6) medical center, (7) community center, (8) insurance company, (9) patient portal, (10) nursing home, (11) staffing agency, and (12) others.
- Educational degree required: Data was sorted for degree required; categories included (1) High school Diploma, (2) Associate Degree, and (3) Bachelor’s Degree.
- Work experience expected: Information was extracted on how much work experience was expected to apply for the various available jobs.
- Location: Using the common way of referring to regions in the US, (27) the location of the vacancies across the US states was also recorded as (1) Northeast, (2) Southeast, (3) Midwest, (4) West, (5) Southwest, (6) Alaska, and (7) remote (anywhere).
- Preferred skills or competencies: Skills and competencies from each job description were coded into 35 categories.

**Figure 2:**
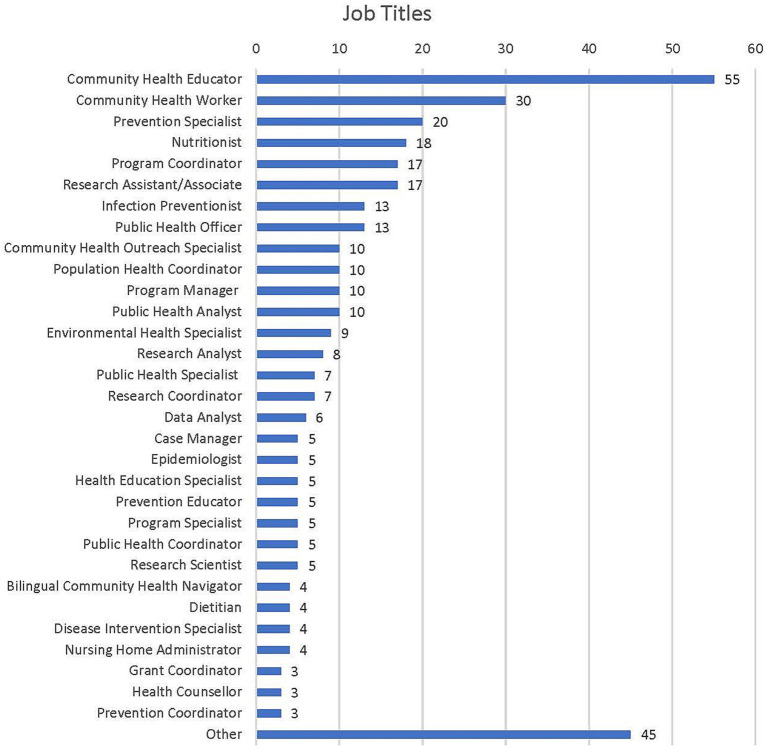
Job titles, frequency (*N* = 365).

Descriptive analysis on 365 postings was performed to report the most common job titles and requirements for the field by frequency and percentage distribution (Tables 1, 2) using Microsoft Excel v.2209 and SPSS v.28. Desired skills listed in job descriptions were extracted from the original spreadsheet and condensed into 35 categories (see Table 3 and Figure 2).

**Table 1:** Job titles (*N* = 365).

Job titles	*n*	*%*
Community Health Educator	55	15.07
Community Health Worker	30	8.22
Prevention Specialist	20	5.48
Nutritionist	18	4.93
Program Coordinator	17	4.66
Research Assistant/Associate	17	4.66
Infection Preventionist	13	3.56
Public Health Officer	13	3.56
Community Health Outreach Specialist	10	2.74
Population Health Coordinator	10	2.74
Program Manager	10	2.74
Public Health Analyst	10	2.74
Environmental Health Specialist	9	2.47
Research Analyst	8	2.19
Public Health Specialist	7	1.92
Research Coordinator	7	1.92
Data Analyst	6	1.64
Case Manager	5	1.37
Epidemiologist	5	1.37
Health Education Specialist	5	1.37
Prevention Educator	5	1.37
Program Specialist	5	1.37
Public Health Coordinator	5	1.37
Research Scientist	5	1.37
Bilingual Community Health Navigator	4	1.10
Dietitian	4	1.10
Disease Intervention Specialist	4	1.10
Nursing Home Administrator	4	1.10
Grant Coordinator	3	0.82
Health Counsellor	3	0.82
Prevention Coordinator	3	0.82
Other	45	12.33

**Table 2:** Sample descriptive statistics (*N* = 365).

Organization type	*n*	*%*
Government	125	34.25
Non-Profit Organization	93	25.48
Educational Institution	32	8.77
Hospital	29	7.95
Healthcare Provider	23	6.30
Staffing Agency	17	4.66
Medical Center	17	4.66
Community Center	14	3.84
Insurance Company	3	0.82
Patient Portal	3	0.82
Nursing Home	3	0.82
Others	6	1.64
**Degree required**		
High school Diploma	50	13.70
Associate Degree	29	7.95
Bachelor’s Degree	286	78.36
**Work experience required**		
Less than 1 year	5	1.37
1 year	76	20.82
1–3 years	85	23.29
4 years and above	12	3.29
Some Experience	119	32.60
Intermediate Experience	1	0.27
Extensive Experience	2	0.55
Not Listed	65	17.81
**Location**		
Northeast	118	32.33
Southeast	86	23.56
Midwest	66	18.08
West	57	15.62
Southwest	27	7.40
Alaska	5	1.37
Remote	6	1.64

**Table 3:** Preferred skills and competencies (*N* = 365).

Skills	*n*	*%*
Public health communication and campaigns - outreach/intervention	337	92.33%
Public health education and promotion	311	85.21%
Cultural competency - sensitivity to population-specific issues	275	75.34%
Project development, implementation, management, and compliance	244	66.85%
Case management skills – referrals and assessments	208	56.99%
Administrative and organizational skills	204	55.89%
Technical writing, grant writing, reports, and manuscripts	167	45.75%
Data collection and analysis	148	40.55%
Software competency – MS Office and data analysis software	134	36.71%
Documentation and reporting of project activities	130	35.62%
Multi-agency/cross-sectoral communication	124	33.97%
Health Program evaluation and improvement	120	32.88%
Collaboration and partnerships	118	32.33%
Behavioral and mental health issues	95	26.03%
Infectious and chronic disease prevention and control	95	26.03%
Health informatics – data management and visualization	72	19.73%
Environmental – health planning, investigation, and assessment	59	16.16%
Health policy development, implementation, and analysis	58	15.89%
Environmental – industrial hygiene and occupational safety	56	15.34%
Epidemiology and disease surveillance	56	15.34%
Public health leadership	46	12.60%
Work with at-risk populations	46	12.60%
Problem solving and critical thinking	43	11.78%
Financial management, budget, grant management	37	10.14%
Literature review and reporting	35	9.59%
Develop and conduct surveys	31	8.49%
Quantitative research methods and statistical analysis	24	6.58%
Disaster preparedness and emergency management	23	6.30%
Develop and implement behavior change intervention plan	19	5.21%
Communication - using media for health promotion	18	4.93%
Food and water safety concerns	18	4.93%
Evidence-based intervention and best practices	18	4.93%
Experience with electronic health records	15	4.11%
Needs assessment	8	2.19%
Qualitative research methods and analysis	7	1.92%

## 3. Results

### 3.1. Sample descriptive statistics

#### 3.1.1. Job titles and organization types

**Figure 3:**
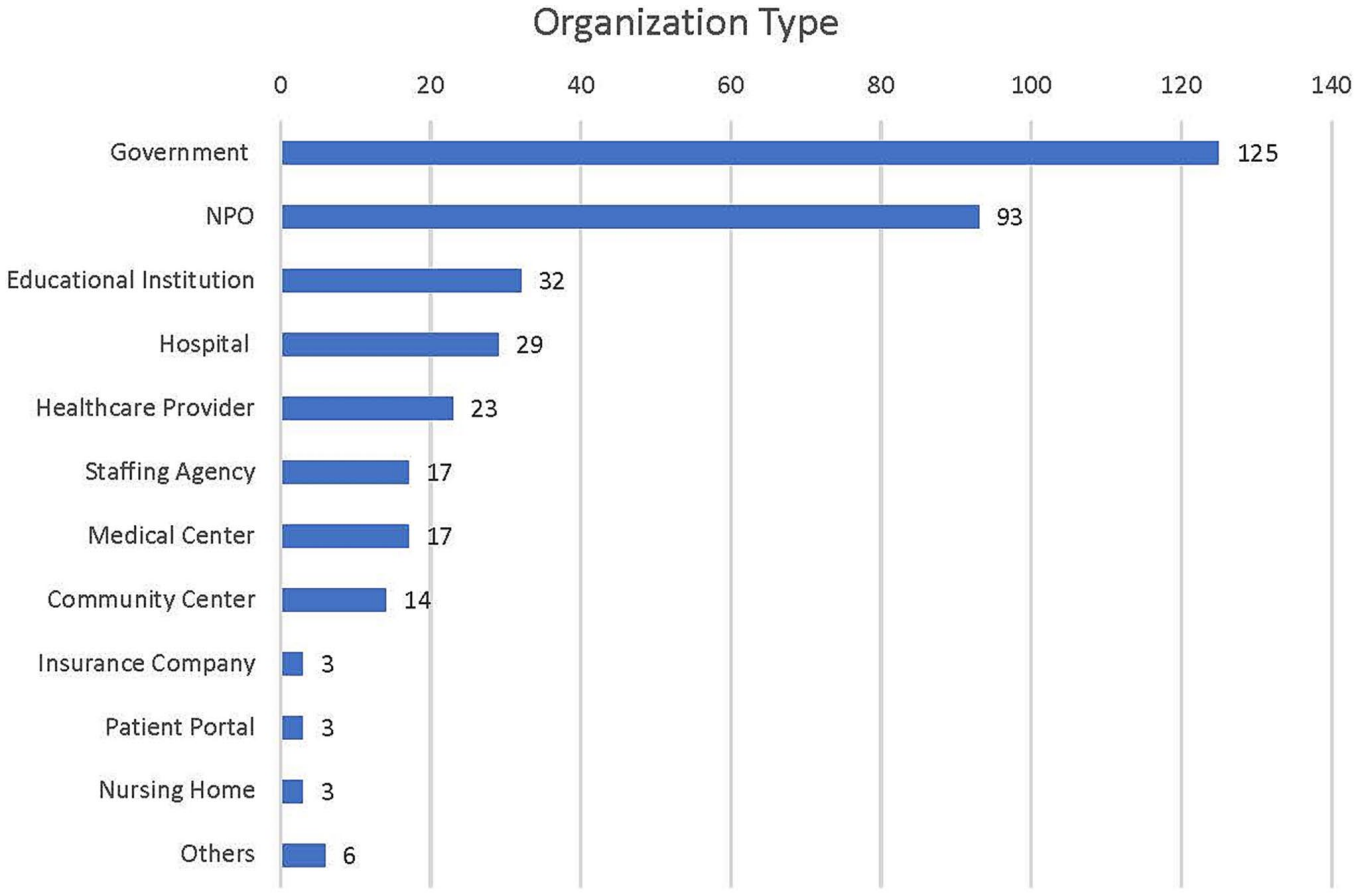
Organization type, frequency (*N* = 365).

In this study’s sample of 365 job postings, 31 job titles were mentioned at notable frequencies. The most frequently posted position (15.07%) was for a Community Health Educator. The second-ranking job title was Community Health Worker (8.22%), followed by Prevention Specialist (5.48%). All other specific job titles were represented by less than 5% of the total postings (see Table 1 and Figure 3). Of the types of organizations posting bachelor-level public health jobs, government organizations were the most represented (34.45%), followed by NPOs at 25.48% (see Table 2). Educational institutions, which accounted for 8.77% of the organization postings, were responsible for most research-related postings (i.e., Research Assistant/Associate, Research Scientist, Research Analyst, and Data Analyst). Though entries that required medical degrees were excluded, 7.95% of public health job postings were at hospitals, 6.30% at healthcare providers, and 4.66% at other medical centers.

#### 3.1.2. Degrees and work experience required

Since a bachelor’s level education was one of the inclusion criteria, the majority of job postings required a bachelor’s degree (78.36%). Some postings requiring only a high school diploma (13.70%) or an associate degree (7.95%) were not screened out because those posts also listed a bachelor’s degree as “preferred.” Nearly one third (32.60%) of job postings required “some experience,” 23.29% sought workers with 1–3 years of experience, and 20.82% required 1 year of experience. Employers did not expect lengthy experience for the positions, as only 3.29% required 4 or more years of experience, and less than 1% each required either “intermediate experience” (0.27%) or “extensive experience” (0.55%) (see Table 2).

#### 3.1.3. Job locations

The geographic distribution of job postings was consistent with regional population densities. For instance, the more densely populated Northeastern region of the US accounted for 32.33% of job posts (118 out of 365). The Southeastern region, with large population centers and rural areas, accounted for 23.56% of postings, followed by the Midwest (18.08%), which is similarly disparately populated. The West and Southwestern portions of the country represented 15.62 and 7.40% of postings, respectively. Five (1.37%) job postings were in Alaska, and six (1.64%) were remote positions (see Table 2).

### 3.2. Preferred skills and competencies

#### 3.2.1. Skills mentioned in more than 50% of job postings

Almost all (92.33%) of job postings required strong communication skills, both oral and written, especially pertaining to public health outreach and intervention. Of nearly equal importance, with 85.21% of postings represented, were community health education and promotion skills. This included the creation and evaluation of educational material to be shared in both broad interventions and for at-risk population groups, as determined by the nature of the organization or project. Cultural competency and sensitivity to population-specific needs were also foremost concerns, as seen in 75.34% of the postings. Some demands of this category of skill included bi- or multi-lingual capabilities and specific knowledge of populations, stage-of-life, and developmental needs, such as maternal-child health and geriatric issues. The capacity to administer a project from development through evaluation was also a highly sought-after skill (66.85%). Mentions of administrative and organizational aptitude were also prevalent (55.89%) outside of the context of project management.

#### 3.2.2. Skills mentioned in between 25 and 50% of postings

Nine skills and competencies were listed by 25–50% of all employers. Technical writing skills, with 167 mentions in the data (45.75%), refer to a higher level of written communication skills, including grant writing as well as the development of project reports and manuscripts. Data collection and analysis were listed in 40.55% of job postings, with data analysis software capability and computer competency close behind (36.71%). The ability to document and report project activities was specified by 35.62% of employers. Cross-sectoral team communication (33.97%) and the capacity to work in multi-agency relationships and collaborations (32.33%) were also listed in this data segment. Health program evaluation was listed in 32.88% of the job postings. Also included were two public health specializations: behavioral and mental health and infectious and chronic disease prevention and control; both were specified in 26.03% of the postings.

#### 3.2.3. Skills listed in between 10 and 25% of job postings

Skills in health informatics, including data management and visualization, were sought in 72 postings (19.73%). Related competencies in epidemiology and disease surveillance were mentioned by 15.34% of employers. Skills in health planning and investigation, as well as industrial hygiene and occupational safety, were included in 16.16 and 15.34% of posts, respectively. Knowledge about policy implementation and analysis was mentioned by 15.89% of employers. Leadership and supervisory competency were noted by 12.60% of employers, as was skill with specific at-risk populations (for instance, incarcerated populations and domestic violence survivors). The phrases “problem solving” and/or “critical thinking” were noted 43 times. Skills related to budgeting, grant management, or other financial competencies were listed in 10.14% of posts.

#### 3.2.4. Skills listed in less than 10% of postings

The ability to conduct a literature review and report findings was explicitly mentioned by 9.59% of employers. This category also included the capacity to develop and conduct surveys (8.49%), quantitative research methods (6.58%), and qualitative research methods (1.92%). Training in disaster preparedness and emergency management was mentioned 23 times (6.30%); there were 19 references (5.21%) to behavior change intervention planning. Experience with mass media for public health promotion was required by 4.93%, as was knowledge of food and/or water safety and competencies with evidence-based interventions and best practices. Fifteen postings mentioned experience with electronic health records (4.11%), and eight (2.19%) referred to “needs assessment.”

## 4. Discussion

In this study, we examined the job skills required at the bachelor’s level by public health employers in the US over the course of 1 week in 2022. This study provides a snapshot of public health workplace skill demands for those with an undergraduate education. The field of public health is constantly evolving, and changes in the field need to be evaluated regularly to track progress in public health trends and ensure that education for public health efforts meets the demands of the workforce.

Results of the study indicate that the WHO’s projection of a healthcare workforce shortage is well-founded (8). More than one third (34.45%) of job postings were from government employers, yet Plepys et al.’s (10) study of first-destination employment for public health graduates found that just 17% of the study participants were going to work for the government. Our study is reflective of late or post-pandemic trends, which may account for a greater demand as many health workers have dealt with various degrees of “burnout” from the stress of the pandemic (7). As Plepys et al.’s (10) data were gathered pre-pandemic, additional research is needed on the nature of this discrepancy. The analysis of PH WINS data, which solely examines data from the governmental public health workforce, indicates that while about 37% of public health workers have only a bachelor’s-level degree, only 14% have a degree at any level with a public health focus (28). Thus, governmental public health employers are hiring individuals without public health training. Identifying the cause of this discrepancy and ascertaining a plan to remedy it is outside the realm of this study, but Locke’s et al.’s (29) examination indicates that dissatisfaction with pay and a lack of opportunity for advancement were the top two reasons workers listed for intending to leave governmental public health positions. Both of these were indicated by more than half of the younger generations of workers (29). A closer examination of economic implications of the governmental public health workplace may be in order to explore this issue further.

Data from our study reinforces the understanding of public health as a multidisciplinary field. As there is no clear majority of job titles represented in the data, we can ascertain that the field of public health includes a broad spectrum of potential employment opportunities. The challenge for universities is to provide the competencies needed in an undergraduate setting for a wide array of jobs. The three skills listed in 75% or more postings were: (1) communication and campaigns (92.33%), (2) education and promotion (85.21%), and (3) cultural competency (75.34%), align with Zemmel et al.’s (22) qualitative study of public health workforce development needs, which found that communication skills were perceived to be the most important in the field. Cultural competency is another important skill that receives attention from many researchers investigating gaps in public health worker training (16, 30), thus, verifying the need for cultural competency and population-specific sensitivity.

Job skill results positively reflect on the National Consortium for Public Health Workforce Development’s specialized and strategic skills model (13). Even if specific terms, such as “systems thinking” and “change management,” were not used in job descriptions, investigation reveals that many of the qualities sought are components of larger skills sets. For instance, “systems thinking” – a set of methodologies that recognizes complexity and interconnectivity and seeks solutions from a variety of perspectives (31) – could be found in data categories such as collaboration and partnerships (listed by 32.33% of employers), multi-agency/cross-sectoral communication (33.97%), public health leadership (12.60%), and project development, implementation, and management (66.85%). Similarly, “change management,” the skills necessary to facilitate organizational change, including leadership, communication, and organizational skills (32), include many of the same categories as “systems thinking” skills.

Other strategic competencies, such as “persuasive communication,” “data analytics,” and “diversity and inclusion” are more easily recognized in the preferred skillset and demanded in over 40% of the job postings. Apart from “health education,” which is listed in 85.21% of job postings, the specialized skills (communicable disease control, chronic disease and injury prevention, environmental public health, epidemiology, and maternal, child, and family health) are narrowly but evenly distributed within the range of preferred skills data. For instance, “epidemiology and disease surveillance” was listed in 15.34% of postings, and the skill category of “infectious and chronic disease prevention and control” was required in 26.03% of postings. Our analysis validates the National Consortium for Public Health Workforce Development’s conclusion that certain skills are useful, regardless of a specific public health focus. We echo the consortium’s call for an increased focus on “strategic skill” competencies in public health education (13).

Two other trends in public health employment met with surprising results in our data analysis. First, studies indicate that public health workers have skill gaps pertaining to financial management (16, 18, 23), with one study finding that up to 55% of public health employees self-report skill gaps pertaining to budgeting (23). Interestingly, financial management and budgeting (including grant management) were only listed in 10.14% of postings at the bachelor’s level. This may indicate that financial skills are being underestimated by employers as they assemble job descriptions, or are not a skill expected at the undergraduate level of public health training (18). Second, while none of the four skill categories related to environmental health or climate change (“food and water safety,” “disaster preparedness and emergency management,” “industrial hygiene and occupational safety,” and “health planning and investigation”) were listed by more than 20% of employers, their representation in the preferred skill list totals 42.73%. Krasna et al.’s (3) study of public health job postings over a span of 16 years posits that this niche is valued, expanding, and could grow rapidly in the coming decade.

Similarly, many recent studies have examined the growing role of health informatics in public health applications (12, 25, 33). Three job titles related to data analysis (“Public Health Analyst,” “Research Analyst,” and “Data Analyst”) accounted for 24 positions. “Health Informatics: data management and visualization” was listed as a required skill in 19.7% of postings, and 40.55% of postings included “data collection and analysis.” Like climate-related public health, informatics is another rapidly-evolving field of study that demands our attention as we anticipate future public health needs (4, 26).

### 4.1. Limitations

As we sought to investigate current public health workforce demands, this study is limited to a small window of time. Therefore, our results are reflective only of the time period for the postings included in this study. Also, just one employment website was used to collect job postings for this study. It is possible that other web postings would have provided a slightly different profile of results. Thus, future studies should expand our search criteria by using multiple sources and a broader data collection period to develop a better understanding of employers’ expectations. Our study was also restricted to job postings within the US, which may limit the relevance of our study for international scholars. This study is of national scope and does not address regional differences in workplace demand. We acknowledge that there could be different skill demands in different regions of the US and hope that this study may inspire further investigation. In addition, this analysis is an exploratory component of an effort to revise our undergraduate public health curriculum to align with the skillset desirable in the current workforce. Therefore, we do not have the outcomes of our curriculum revisions available at this point.

### 4.2. Public health implications

Our goal in this study was to examine the skill demands of the current public health workplace for those with a bachelor’s degree and to ascertain the desired skills and competencies sought by public health employers in the US. Results revealed the most sought-after skillset required in the current public health job market and may give higher education administrators additional evidence necessary to align their curricula to adequately prepare students for the public health workforce. In the multidisciplinary field of public health, it remains critical for educators to consider real-world workplace demands (34). An honest evaluation of the industry requirements and training gaps will fuel critical changes in the undergraduate curricula and important updates in the competencies to train the future public health workforce. This detailed evaluation of a slice of the public health labor marketplace for undergraduate-level jobs, with a focus on the skills and competencies most demanded by employers, will contribute to on-going efforts to reform public health education at the undergraduate level and lead to a healthier future for our communities, nation, and world.

## Data availability statement

The raw data supporting the conclusions of this article will be made available by the authors, without undue reservation.

## Author contributions

AJ and SK conceived and designed this study. GL collected and collated the data. SK and CE sorted and coded the data, then wrote the first draft of the manuscript. AJ, LM, and EB contributed to further thematic development of the manuscript. All authors made substantial contributions to the revision of the manuscript, read and approved the submitted version.
